# Transcriptomics and microbiome insights reveal the protective mechanism of mulberry-derived postbiotics against inflammation in LPS-induced mice

**DOI:** 10.3389/fimmu.2025.1536694

**Published:** 2025-02-18

**Authors:** Zaheer Abbas, Yucui Tong, Jing Zhang, Abdul Sammad, Junyong Wang, Baseer Ahmad, Xubiao Wei, Dayong Si, Rijun Zhang

**Affiliations:** ^1^ State Key Laboratory of Animal Nutrition and Feeding, College of Animal Science and Technology, China Agricultural University, Beijing, China; ^2^ Key Laboratory of Animal Genetics, Breeding and Reproduction, College of Animal Science and Technology, China Agricultural University, Beijing, China; ^3^ Faculty of Veterinary and Animal Science, Muhammad Nawaz Sharif University of Agriculture, Multan, Pakistan

**Keywords:** mulberry-derived postbiotics, lipopolysaccharides, transcriptomics, gut microbiota, immune response

## Abstract

**Background:**

Natural food-derived bioactive compounds have garnered increasing attention for their potential to modulate immune responses and promote gut health. In particular, compounds like mulberry-derived postbiotics (MDP) may offer novel therapeutic strategies to address inflammation, a key driver of many metabolic disorders.

**Methodology:**

This study examines the protective effects of MDP against inflammation in LPS-induced mice, using transcriptomic and microbiome analyses to explore underlying mechanisms.

**Results:**

MDP pretreatment alleviates LPSinduced villous atrophy and intestinal barrier damage, promoting recovery of intestinal morphology. Transcriptomic profiling revealed significant changes in gene expression, with 983 upregulated and 1220 downregulated genes in the NC vs LPS comparison, and 380 upregulated and 204 downregulated genes in the LPS vs LPS+MDP comparison. Enrichment analysis using GO and KEGG pathways revealed significant associations with transcriptional regulatory activity, and the NOD-like receptor signaling pathway among the differentially expressed genes. Protein-protein interaction analysis identified key genes involved in inflammation and immune regulation, with hub genes like IL6, CXCL10, and MYD88 in the LPS group and CD74, CIITA, and H2-AB1 in the MDP-treated group.

**Conclusion:**

Microbiome analysis suggested MDP may also influence gut microbiota composition, supporting systemic immune regulation. These findings highlight MDP’s potential as a food additive for immune modulation and gut health.

## Graphical Abstract

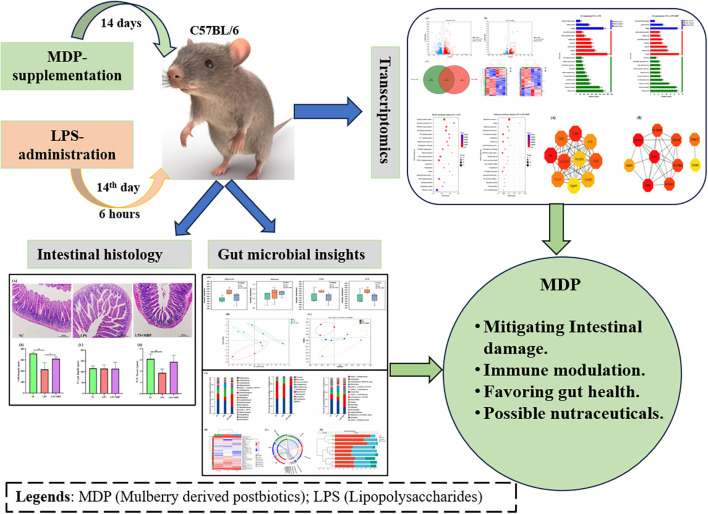

## 1 Introduction

In recent years, there has been a burgeoning interest in exploring natural compounds and their derivatives for their potential therapeutic benefits against oxidative stress and inflammation, which are implicated in various chronic diseases and aging processes (1, 2). Among these natural sources, mulberry (Morus spp.) has gained attention not only for its nutritional value but also for its bioactive components that possess antioxidant and anti-inflammatory properties (3, 4). Mulberry-derived postbiotics, including peptides and other metabolites produced during fermentation, have emerged as promising candidates in this regard (5–7). Studies have shown that these bioactive compounds can mitigate oxidative damage and suppress inflammatory responses through various mechanisms, including modulation of cellular signaling pathways and regulation of gene expression (8, 9).

Inflammation is a fundamental immune response to harmful stimuli, such as pathogens, injury, or toxins, and plays a key role in tissue repair and host defense (10, 11). Acute inflammation is typically a protective mechanism aimed at neutralizing the harmful agent and initiating the healing process. This response is characterized by the release of inflammatory mediators like cytokines, prostaglandins, and reactive oxygen species (ROS), which help to contain the threat and promote tissue repair (12, 13). However, when inflammation becomes chronic, it can lead to sustained tissue damage and contribute to the pathogenesis of various diseases, including autoimmune disorders, cardiovascular disease, and cancer (14, 15). Chronic inflammation is often driven by the persistent activation of immune cells such as macrophages, neutrophils, and T-cells, which release pro-inflammatory cytokines that maintain the inflammatory cycle (16, 17). One critical aspect of inflammation is oxidative stress, a condition where there is an imbalance between ROS production and antioxidant defenses. ROS, produced by activated immune cells during inflammation, can further damage tissues and activate inflammatory pathways, leading to a vicious cycle of inflammation and oxidative damage (18–20). The relationship between inflammation and oxidative stress is pivotal in the progression of many chronic inflammatory diseases (10, 18, 21), highlighting the need for therapeutic strategies that target both inflammatory mediators and oxidative damage.

Postbiotics refer to the bioactive compounds produced during the fermentation of probiotic bacteria or by the enzymatic activity of these bacteria on substrates such as mulberry (22, 23). These compounds include metabolites such as short-chain fatty acids, peptides, polysaccharides, and other biologically active molecules (24, 25). Unlike probiotics, which are live microorganisms, postbiotics offer the advantage of stability and safety, making them easier to incorporate into various applications including functional foods, dietary supplements, and pharmaceuticals (26, 27). Mulberry-derived postbiotics have attracted attention due to their potential health benefits beyond traditional probiotics. Studies have demonstrated that these postbiotics possess antioxidant, anti-inflammatory, antimicrobial, and immunomodulatory properties, suggesting a broad spectrum of therapeutic applications (5, 28). For instance, peptides derived from mulberry fermentation have been shown to exhibit antioxidant activity by scavenging free radicals and enhancing the activity of antioxidant enzymes such as superoxide dismutase (SOD) and catalase (29, 30). Recent studies have further highlighted the therapeutic potential of postbiotics in models of gut inflammation, particularly in mice with LPS-induced inflammation. Postbiotics such as those derived from mulberry through fermentation have shown promising effects in mitigating gut inflammation by regulating immune responses and promoting gut microbiome balance (28). These studies suggest that postbiotics could help restore gut homeostasis, reduce inflammatory cytokines production, and enhance epithelial barrier function, offering a novel therapeutic approach to inflammatory bowel diseases and other chronic gut conditions.

Transcriptomic analysis, which allows for a comprehensive assessment of gene expression patterns in response to treatment, provides a powerful tool to elucidate the molecular mechanisms underlying these protective effects (31, 32). In the context of studying the protective mechanisms of mulberry-derived postbiotics against oxidative stress and inflammation, transcriptomic analysis offers several advantages. Transcriptomics data encapsulates vital information regarding gene expression activities within specific cells, tissues, or populations under particular developmental stages, environmental conditions, or experimental conditions (32, 33). Unlike other omics data, transcriptomics data uniquely reflects the temporal and spatial variations influenced by both diverse internal and external environmental factors, making it inherently more complex than genomic data. It allows researchers to identify differentially expressed genes (DEGs) in response to treatment, uncover potential biomarkers of oxidative stress and inflammation, and elucidate the molecular pathways through which mulberry-derived postbiotics exert their effects. Furthermore, transcriptomic data can facilitate the discovery of novel therapeutic targets and guide the development of targeted interventions for oxidative stress-related diseases (34, 35). In parallel, recent studies have highlighted the important role of the gut microbiome in modulating inflammatory responses (36–38). In models of LPS-induced inflammation, alterations in microbial diversity are often observed, with shifts towards dysbiosis being linked to the exacerbation of inflammation (39, 40). Specifically, the reduction in beneficial microbes such as Firmicutes and an increase in pathogenic bacteria have been reported in inflammatory conditions, further aggravating the inflammatory process (41, 42). These findings underscore the importance of understanding how microbial communities can influence the inflammatory process, and how therapeutic strategies, such as the use of postbiotics, may potentially restore microbial balance and mitigate inflammatory responses. Moreover, microbial diversity studies will provide crucial insights into how the gut microbiome responds to mulberry-derived postbiotics (MDP), revealing shifts in microbial populations that may enhance the anti-inflammatory and antioxidative properties of these postbiotics. A deeper understanding of the relationship between microbial diversity and host gene expression could further inform the development of postbiotics-based therapies that synergistically modulate both the immune system and the gut microbiota to combat inflammation.

Therefore, by combining transcriptomics analysis with microbiome profiling, this study aims to uncover the molecular mechanisms by which MDP interact with the host immune system and gut microbiota to provide a multifaceted protective effect against LPS-induced inflammation thereby providing a deeper understanding of their therapeutic potential. Furthermore, this integrated approach will offer new insights into the therapeutic potential of MDP in regulating the microbiome-immune axis and mitigating chronic inflammatory diseases.

## 2 Materials and methods

### 2.1 Production of MDP

Mulberry-derived postbiotic (MDP) was prepared by fermenting mulberry leaves powder medium composed of 1 g of glucose, 0.1 gram of K_2_HPO_4_.3H_2_O, and 0.05% MgSO_4_.7H_2_O. The mixture was sterilized at 121°C for 15 minutes, after which bacterial cultures of *Bacillus subtilis* H4 and *Bacillus amyloliquefaciens* LFB112 were added and incubated at 37°C for 24 h. The optimized conditions for this process are detailed in our prior publication (5). Following fermentation, the broth was centrifuged at 5000×g for 10 min, and the supernatant was filtered using 0.22 µm filter paper. The filtrate was then freeze-dried, and the composition of the postbiotics was analyzed using advanced instrumental and analytical methods. The resulting MDP powder had a dry matter content of 85.35%, with a moisture content of 14.65%, crude fiber (0.01%), total protein (151.32 mg/g), with soluble protein (149.76 mg/g), and total carbohydrate content of 324.91 mg/g of (expressed in glucose).

### 2.2 Animals and MDP dosage

A total of 36 C57BL/6 mice (weighing 20-22 g and aged 6-8 weeks) were acquired from Jiangsu Jicui Yakong Biotechnology Co., Ltd. (Beijing, China). The mice were housed in a controlled environment (temp: 21-23°C, relative humidity: 40-70%), with a 12h light-dark cycle). All procedures involving the animals adhered to the experimental animal welfare standards approved by the Animal Use and Care Committee of China Agricultural University. After a week of acclimation, the mice were randomly assigned to three groups each consisting of 12 mice: control group, LPS group, and LPS+MDP group. The control group was free of LPS and MDP, the LPS group was only administered LPS at a dose of10 µL (2 µg/g body weight) via intraperitoneal injection. In the LPS+MDP group, MDP was administered orally at a dose of 100 mg/kg body weight and LPS with the same dosage as the LPS group.

### 2.3 Histology of the jejunum tissue

The jejunum tissue was fixed with 4% paraformaldehyde, and dehydrated with ethanol. Subsequently, xylene was used for equilibration and the tissue was embedded in paraffin. A tissue slicer was employed to cut tissue sections to a thickness of around 5 µm. The sections were then stained with hematoxylin and eosin (H&E) stains and were observed under a light microscope and analyzed using ImageJ image analysis software.

### 2.4 Gut microbiota analysis

Fecal samples were processed to extract genomic DNA using the QIAmpR Fast DNA Stool Mini Kit (Qiagen Ltd., Hilden, Germany), following the manufacturer’s instructions. The V3 and V4 region of the 16S rRNA gene was then amplified using the primers 338F (5′∼3′: ACTCCTACGGGAGGCAGCAG) and 806R (5′∼3′: GGACTACHVGGGTWTCTAAT). After amplification and purification, the products were combined in equal molar concentrations and sequenced on an Illumina HiSeq 2500 platform, generating 300 bp paired-end reads. Sequence variants in the amplified V3-V4 region were identified using the same primers. Data analysis was conducted using the Majorbio Cloud Platform (www.majorbio.com).

### 2.5 RNA extraction

RNA was extracted from Jejunum samples utilizing the TRIzol Reagent method, adhering to the manufacturer’s protocol. RNA quality was assessed with a 5300 Bioanalyzer (Agilent, Invitrogen, California, USA) and quantified using a Nanodrop-2000 spectrometer (Thermo, Massachusetts, USA). RNA samples meeting the following criteria were used for library construction: OD260/280 ratios of 1.8-2.2, OD260/230 ≥2, RQN ≥6.5, and a minimum concentration of 1 µg. RNA-seq library was prepared following the Illumina^®^ Standard mRNA prep, ligation protocol using 1µg of total RNA. Subsequently, cDNA was synthesized using a SuperScript cDNA synthesis kit (Invitrogen CA, USA). Size selection for cDNA fragments targeted at 300 bp was performed on a 2% Low Range Ultra Agarose gel, followed by PCR amplification using Phusion DNA polymerase (NEB) for 15 cycles. After quantification with a Qubit 4.0, the sequencing library was sequenced on the NovaSeq X Plus platform (PE150) using a NovaSeq Reagent Kit.

### 2.6 Quality assessment and read alignments

The quality of the sequencing read was assessed using FastQC software (v0.12.0). Subsequently, all reads were filtered to remove adapters, short sequences, low complexity reads, and low quality data, along with trimming, using Fastp software (v0.20.0). Cleans reads were then aligned to the reference genome of Mus_musculus (http://asia.ensembl.org/Mus_musculus/Info/Index) and the genes with matching rates ranging from 97.91 to 98.09% were selected.Three cDNA libraries were generated from the mRNA extracted from the jejunum tissue of C57LB/6 mice in the NC, LPS, and LPS+MDP groups, and these libraries were sequenced using the Illumina platform. After filtering out low-quality sequences and contaminants, the total raw reads averaged approximately 52001846.67 base pairs, as shown in **Table 1**. The filtering process resulted in an average of 48486813.99 clean reads. Specifically, the NC group produced an average of 54243632.67 high-quality reads from 54943621.33 raw reads, the LPS group yielded 46603170.67 clean reads from 47307653.33 raw reads, and the LPS+MDP group had an average of 53778972 reads from 54168265.33 raw reads. All the groups achieved Q20% above 98% and Q30% exceeding 96%, indicating high quality in the sequencing data. The assembly results confirm that the sequencing was of sufficient quality for transcriptomics analysis.

**Table 1:** Transcriptome sequencing in C57LB/6 mice and the statistics of the transcriptome libraries.

Sample	Raw reads	Raw bases	Clean reads	Clean bases	Error rate (%)	Q20 (%)	Q30 (%)	GC content (%)
N1	55223504	8.34E+09	54672262	8.169E+09	0.012	98.74	96.07	48.05
N2	48137712	7.27E+09	47704424	7.131E+09	0.0119	98.77	96.15	47.77
N7	60769648	9.18E+09	60156212	8.987E+09	0.012	98.74	96.09	47.83
P3	50990090	7.7E+09	50521504	7.552E+09	0.0119	98.77	96.13	47.6
P4	47096430	7.11E+09	46634650	6.98E+09	0.0119	98.79	96.22	47.69
P11	43036440	6.5E+09	42653358	6.37E+09	0.012	98.72	96.03	47.27
H5	51746178	7.81E+09	51242066	7.66E+09	0.012	98.75	96.1	47.75
H9	50174862	7.58E+09	49699270	7.432E+09	0.0119	98.78	96.2	47.85
H11	60983756	9.21E+09	60395580	9.031E+09	0.0119	98.76	96.13	47.71

### 2.7 Differentially expressed genes and its functional analysis

DEGs (differential expression genes) were identified between two samples by calculating transcript expression levels using the transcripts per million reads (TPM) technique. RSEM was used to measure gene abundance. Differential expression analysis was done with DESeq2. DEGs with log2FC ≥1 and FDR < 0.05 (DESeq2) were considered substantially expressed genes.

### 2.8 GO and KEGG analysis

Gene ontology (GO) and Kyoto Encyclopedia of Genes and Genomes (KEGG) pathway enrichment analyses were performed to explore the possible functions of differentially expressed genes. In this way, the function of DEGs was classified according to the GO and KEGG. Furthermore, all the DEGs were associated with GO and KEGG terms. After being compared with the genome background via Fisher’s exact test, the significantly enriched GO and KEGG terms were selected for further investigation. Go tools and Python scipy software, were utilized to visually display the significantly changed pathways and their enriched genes through comparison.

### 2.9 Protein-protein interaction

The string online database offers reliable information regarding protein-protein interactions (PPI). In our study, the minimum interaction score for the PPI network set was 0.4. once the PPI information was extracted, Cytoscape was employed for network visualization. Cytoscape is an open-access software designed to visualize complex networks and their interaction with various types of variables.

### 2.10 Validation of genes by quantitative real-time PCR

RNA was extracted from the jejunum tissue using the Triazole reagent as per the manufacturer’s instructions. The integrity of the RNA was evaluated using gel electrophoresis and nanodrop. RNA was reverse transcribed into complementary DNA (cDNA) following the instructions of the Master mix kit. PCR was conducted using the M5-Hiper SYBER Premix EsTaq from Bimake (USA) kit following the manufacturer’s instructions. The amplification process was conducted by starting with an initial preincubation for 30 s at 95°C, and denaturation afterward at 95°C for 5 s, extension at 60°C for 30 s, and finally melting at 95°C for 1 s, 60°C for 1 s, and again 95°C for 1s. The data were analyzed using the 2- ΔΔCt method, with normalization against β-actin. The primers used are listed in **Supplementary Table S1** and were provided by Sangon Biotech Co., Ltd, Shanghai, China.

### 2.11 Statistical interpretation

Transcriptomics and microbial data were analyzed using the Majorbio website (https://cloud.majorbio.com/page/project/overview.html accessed on 25.10.2024). The *P-*value was determined using a one-way analysis of variance (ANOVA). Data was represented as mean ± standard and visualized using GraphPad prism. A *P-*value less than 0.05 was regarded as statistically significant.

## 3 Results

### 3.1 Intake of MDP promoted intestinal repair

**Figure 1:**
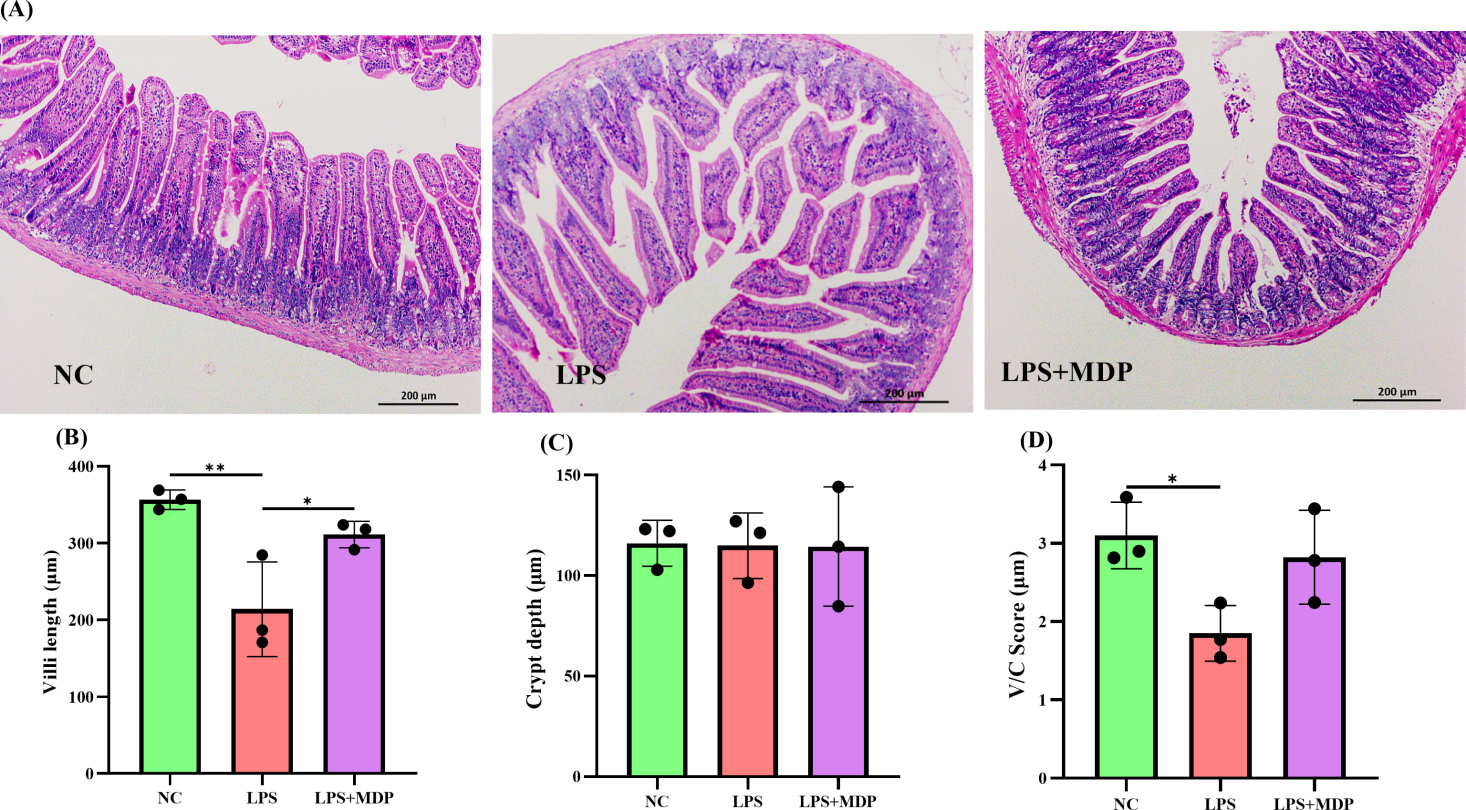
**(A)** Representative H&E staining images of jejunum tissue from NC, LPS, and LPS+MDP groups. **(B)** Villi length measurements in the jejunum. **(C)** Crypt depth analysis in the jejunum. **(D)** Villi to crypt ratio (V/C) in the jejunum. Data are expressed as means ± standard deviation (SD) from three independent experiments (n=3). Statistical significance was determined using Student’s t-test for comparisons between the groups, with p-values indicated as follows: **P < 0.01, *P < 0.05.

Histological examination of the jejunum revealed marked intestinal damage in the LPS-treated group, characterized by significant villous atrophy and disruption of the intestinal barrier integrity. Specifically, LPS treatment led to a reduction in villus height, crypt depth, and the villus-to-crypt (V/C) ratio, all of which are key indicators of intestinal health and barrier function (**Figures 1A–D**). These changes suggest that LPS exposure induced a severe inflammatory response, impairing the structural integrity and regenerative capacity of the intestinal epithelium. To provide a comprehensive view of the tissue features, low-magnification H&E-stained images have been included in the **Supplementary Material** (**Supplementary Figure S1**).

In contrast, pretreatment with mulberry-derived postbiotics (MDP) in the LPS+MDP group significantly ameliorated the LPS-induced damage. MDP administration led to a restoration of normal intestinal morphology, with pronounced improvements in both villus height (**Figure 1B**) and crypt depth (**Figure 1C**). The villus-to-crypt ratio (**Figure 1D**), a critical parameter for assessing the balance between epithelial cell proliferation and differentiation, was also significantly enhanced in the MDP-treated group compared to the LPS-only group. These findings suggest that MDP not only mitigates the detrimental effects of LPS but also promotes the recovery of intestinal architecture, which is essential for maintaining mucosal integrity and function.

### 3.2 Modulation of gene expression by LPS and MDP treatment

**Figure 2:**
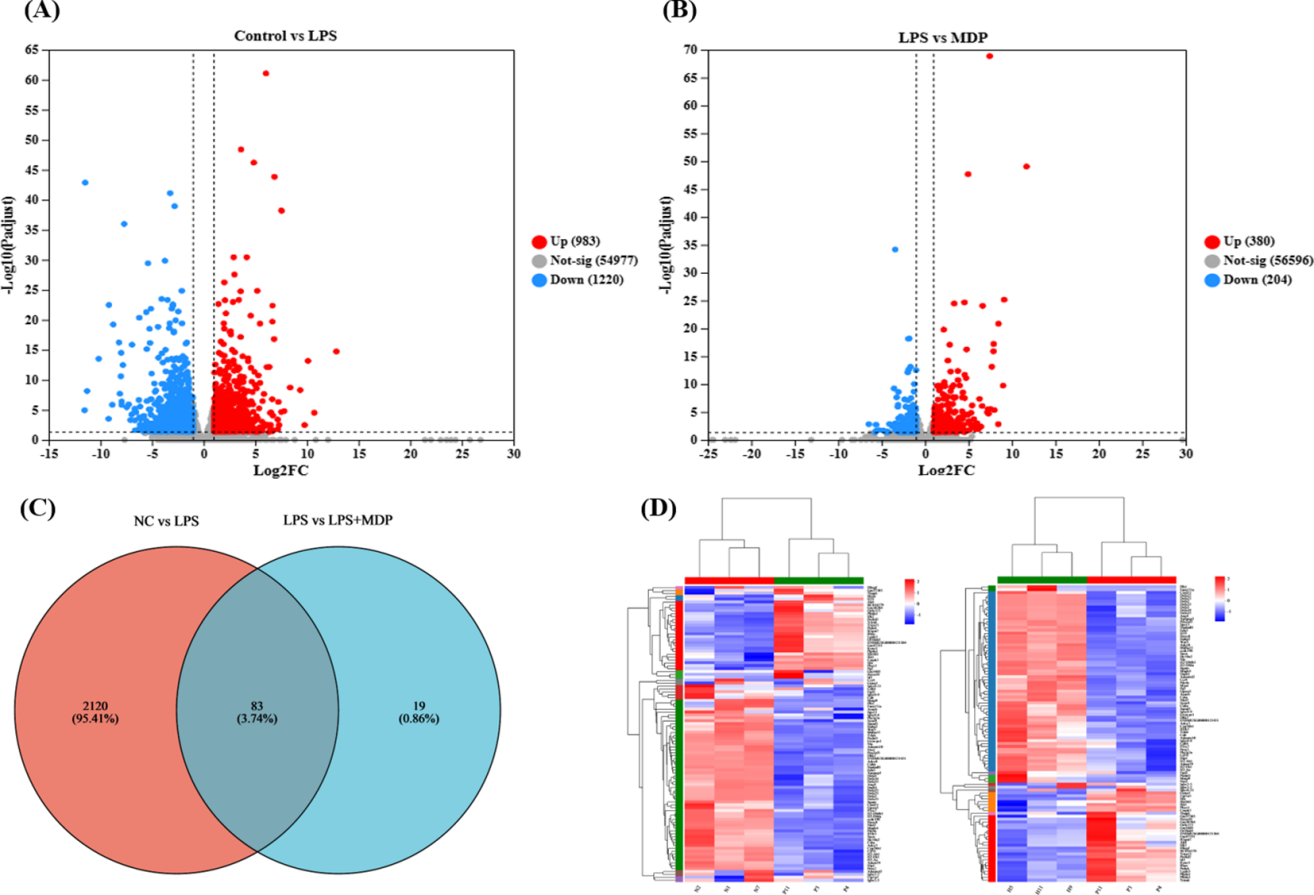
Insights into RNA sequence results. The volcano plot of RNA-seq results presented for the NC *vs* LPS group shows the upregulated and downregulated genes **(A)**, and LPS *vs* LPS+MDP **(B)**. The blue dots represent the down-regulated genes while the red dots represent the up-regulated genes. The horizontal line shows *p* < 0.05, and the vertical line represents absolute log_2_ fold-change (FC ≥ 2) The Venn plot shows the number of overlapping and unique DEGs in each comparison **(C)** and the heat map shows the cluster of the DEGs **(D)**.

The differential gene expression profiles across the experimental groups were visualized through a volcano plot (**Figure 2**), which highlights the significantly upregulated and downregulated genes in each comparison. In the comparison between the negative control group (NC) and the LPS-treated group (NC *vs* LPS), a total of 983 genes were upregulated, while 1220 genes were downregulated (**Figure 2A**). This suggests that LPS treatment induces a broad and significant alteration in gene expression, consistent with its known inflammatory effects. In the comparison between the LPS and the mulberry-derived postbiotic treatment group (LPS *vs* LPS+MDP), the data revealed 380 upregulated genes and 204 downregulated genes (**Figure 2B**). These gene expression changes provide valuable insights into the molecular mechanisms underlying LPS-induced inflammation, which serves as a model for understanding the severity of intestinal damage.

A Venn diagram (**Figure 2C**) further elucidates the overlap and distinct sets of differentially expressed genes (DEGs) between the three groups. Specifically, 2120 DEGs were identified in the NC *vs* LPS comparison, while 19 DEGs were observed in the LPS *vs* LPS+MDP comparison. Importantly, 83 DEGs were common in both comparisons, suggesting that these genes are potentially critical in the response to LPS and the modulation by MDP treatment. These shared genes may represent key regulatory pathways involved in the inflammatory process and the recovery of intestinal function. These pathways could be the focus of further investigation to determine the specific molecular mechanisms by which MDP mitigates LPS-induced intestinal inflammation. Furthermore, cluster analysis represented by a heatmap was performed on the DEGs from each comparison (**Figure 2D**). The clustering results demonstrated that genes with similar expression patterns within each group tended to cluster together, confirming the consistency of the data across biological replicates. The cluster analysis strengthens our understanding of how MDP influences gene expression, providing a clearer picture of its potential to restore intestinal homeostasis in the context of LPS-induced inflammation.

### 3.3 Identification of key biological pathways through GO Enrichment analysis

**Figure 3:**
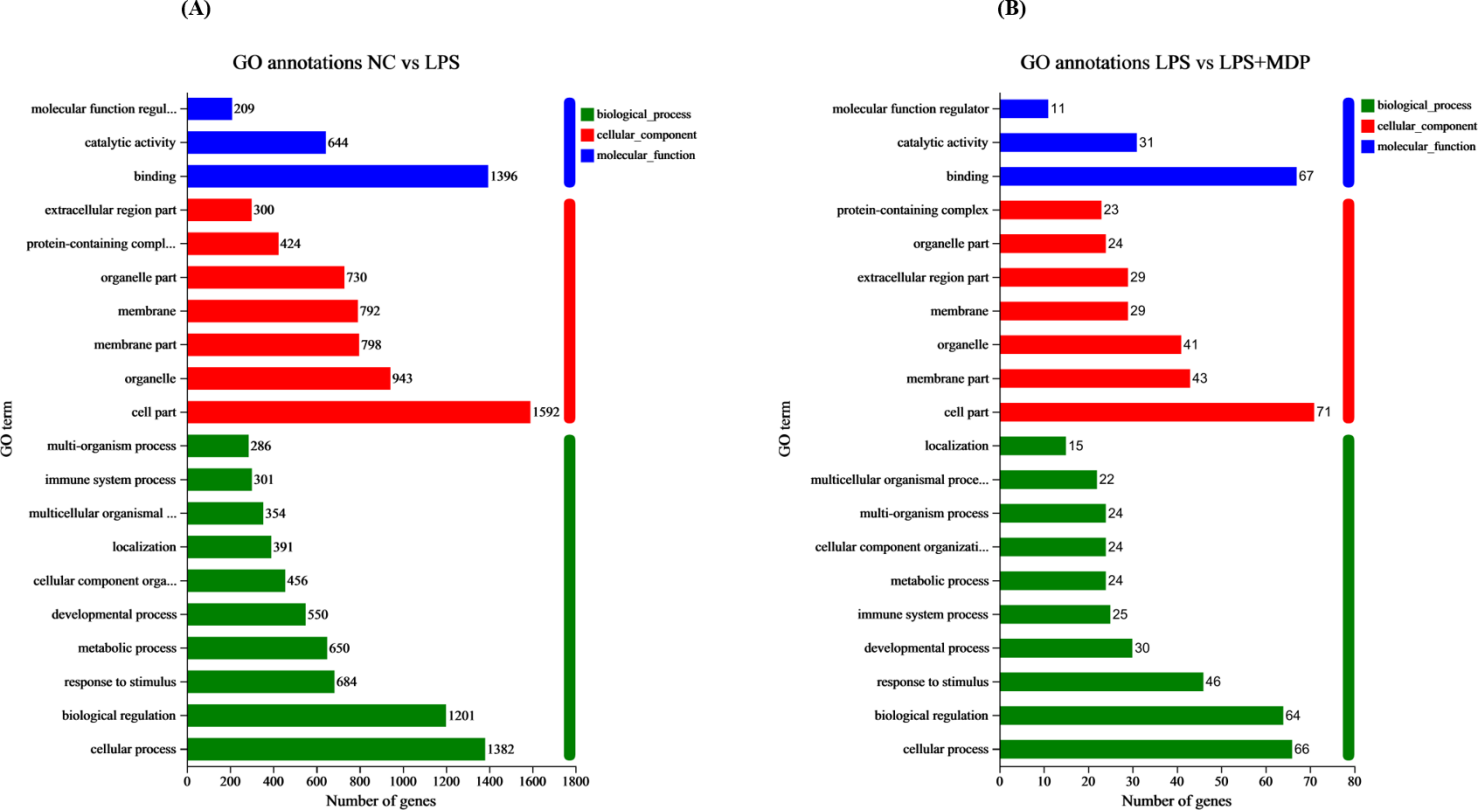
GO terms annotations of the DEGs in the NC *vs* LPS **(A)** and LPS *vs* LPS+MDP group **(B)**. The figure displays the GO terms that are significantly enriched (*p*<0.05). The abscissa corresponds to the terms in GO categories, including biological processes (BP), cellular components (CC), and Molecular Functions (MF). Each rectangular shape represents the most significant GO terms, and the numbers indicate the genes in each GO term.

A Gene Ontology (GO) functional enrichment analysis was conducted on the differentially expressed genes (DEGs) within NC *vs* LPS and LPS *vs* LPS+MDP groups. The differentially expressed genes (DEGs) were categorized into three main Gene Ontology (GO) functional groups: biological process (BP), cellular component (CC), and molecular function (MF), based on their annotations. As shown in **Figure 3A**, when comparing the NC group to the LPS-treated group, enrichment was observed in several key biological processes within the BP category. These included processes such as cellular signaling, biological regulation, immune system response, and responses to stimuli. In the MF category, the DEGs predominantly represented activities related to binding, catalytic activity, and molecular function regulation. In **Figure 3B**, we present the comparison between the LPS and LPS+MDP groups. In the Biological Process (BP) category, the top three enriched pathways were common for both the NC *vs*. LPS and LPS *vs*. MDP comparisons. This suggests that both treatments induce similar core immune processes. The consistency in enriched pathways indicates that the addition of MDP to LPS does not alter the key biological processes activated by LPS alone, such as immune system processes, cellular responses to stimuli, and biological regulation. This result implies that MDP likely modulates or fine-tuned the immune response rather than introducing entirely new biological processes. This suggests that MDP may primarily act as a modulator of the immune system rather than as a direct inducer of new inflammatory processes. Such analysis could reveal specific molecular targets or signaling pathways that MDP acts upon to attenuate the severity of LPS-induced intestinal inflammation. However, a deeper analysis of additional DEGs and downstream pathways may uncover more subtle effects of the combined treatment, providing further insights into how MDP influences the immune response alongside LPS.

### 3.4 KEGG pathway modulation by LPS and MDP treatment

**Figure 4:**
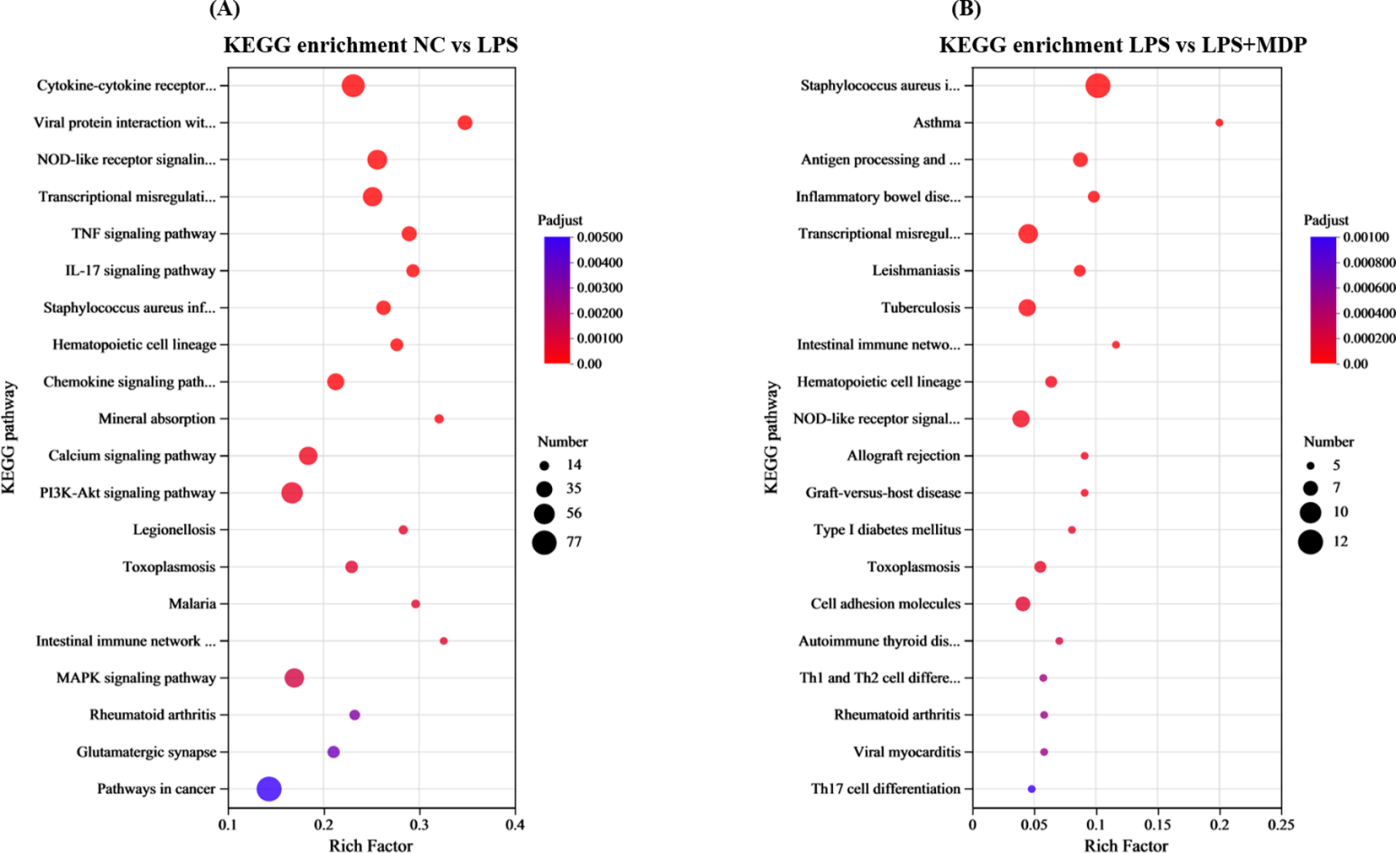
KEGG enrichment pathways of the DEGs in the NC *vs* LPS group **(A)** and LPS *vs* LPS+MDP group **(B)**. The X-axis represents the enrichment factor, while the Y-axis lists the name of the enriched pathways. The size of each dot reflects the number of genes associated with each pathway, and the color of the dots indicates the significance level, with the corresponding *p-*value as shown in the figure legend.

A Kyoto Encyclopedia of Genes and Genomes (KEGG) pathway analysis was carried out on the DEGs in the related groups. The top 20 KEGG pathways for NC *vs* LPS and LPS *vs* LPS+MDP groups are presented in **Figure 4**. The top 20 enriched pathways were determined by performing KEGG functional enrichment analysis on the differentially expressed genes (DEGs) in the NC *vs* LPS and LPS *vs* LPS+MDP comparisons. In the NC *vs* LPS group, pathways such as the cytokine-cytokine receptor interaction, NOD-like receptor signaling, transcriptional misregulation, and the PI3K-Akt signaling pathway were significantly enriched (**Figure 4A**). These pathways underscore the critical involvement of immune signaling, cellular stress responses, and altered transcriptional regulation, which collectively drive the inflammation and tissue damage associated with LPS-induced intestinal injury. In contrast, the LPS *vs* LPS+MDP group showed significant enrichment in pathways related to Staphylococcus aureus infection, transcriptional misregulation, NOD-like receptor signaling, and antigen processing and presentation (**Figure 4B**). While many of the pathways activated by LPS remained prominent, the addition of MDP influenced the dynamics of immune modulation. Notably, the enrichment in antigen processing and presentation pathways suggests that MDP may enhance the adaptive immune response, potentially facilitating a more targeted resolution of inflammation.

### 3.5 Mapping protein-protein interaction of the LPS and MDP-influenced DEGs

**Figure 5:**
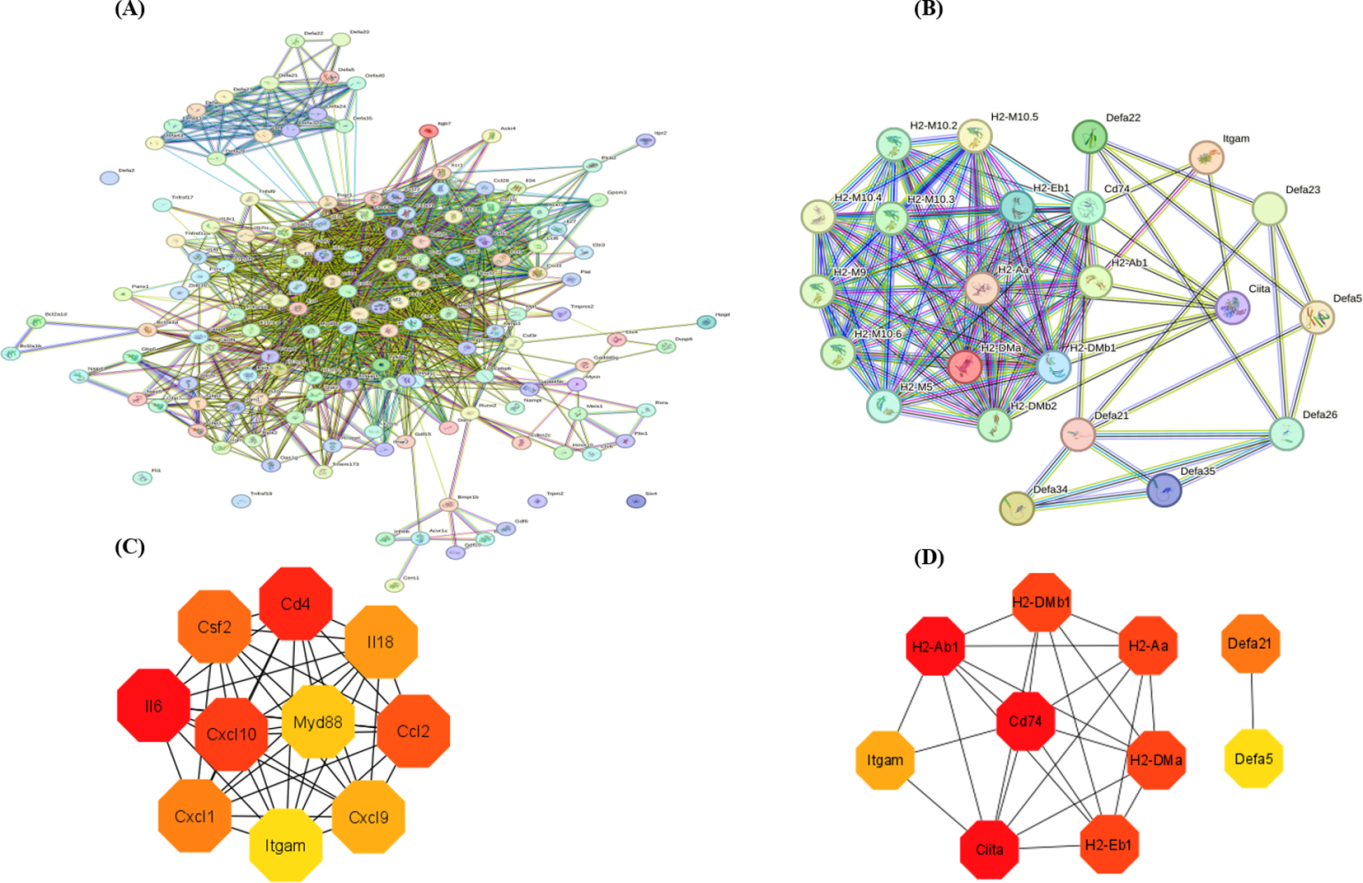
The protein-protein interaction (PPI) network analysis was performed on the most enriched pathways in the NC *vs* LPS and LPS *vs* LPS+MDP groups **(A, B)**. The interaction was performed with a confidence score of 0.9. Top 10 hub genes in the NC *vs* LPS group **(C)**, and in the LPS *vs* LPS+MDP groups extracted from the PPI network **(D)**.

The protein-protein interaction (PPI) network analysis was performed on the differentially expressed genes (DEGs) from enriched pathways between the NC *vs* LPS and LPS *vs* LPS+MDP groups using the STRING database, with a medium confidence score (0.4), as shown in the **Figure 5**. The cytoHubba plugin in Cytoscape 3.9.1 was used to determine the hub genes within the regulatory network. PPI networks were constructed based on data from the STRING database. In the NC *vs* LPS group, a total of 169 genes from three KEGG-enriched pathways were selected, while in the LPS *vs* LPS+MDP group, 31 KEGG-enriched genes in the pathways related to inflammation and immune response were chosen to map the PPI network using the STRING database (http://string-db.org, accessed on 5 November 2024) (**Figures 5A, B**). The PPI network for the NC *vs* LPS group consisted of 143 nodes and 1,442 edges, while the LPS *vs* LPS+MDP group had 35 nodes and 301 edges. Using cytoHubba, the top 10 hub genes were identified in each group. For the NC *vs* LPS group, the hub genes included *IL6, CD4, CXCL10, CCL2, CSF2, CXCL1, IL18, CXCL9, MYD88*, and *ITGAM* (**Figure 5C**), while the hub genes in the LPS *vs* LPS+MDP group included *CD74, CIITA, H2-AB1, H2-DMA, H2-AA, H2-DMB1, H2-EB1, DEFA21, ITGAM*, and *DEFA5* genes (**Figure 5D**). The prominence of genes involved in antigen processing and presentation (H2-AB1, CIITA, and others) reflects the potential role of MDP in enhancing adaptive immunity, suggesting that MDP may fine-tune the immune response by promoting antigen recognition and presentation. The presence of ITGAM in both groups points to its role in modulating immune cell adhesion and migration, a key factor in controlling inflammation. These findings suggest that MDP influences the immune network by enriching pathways related to antigen presentation, highlighting its potential to refine adaptive immune responses. Furthermore, the important information related to these genes is summarized in **Table 2**.

**Table 2:** The top 10 important hub genes with the relative information from the datasets.

Top 10 hub genes of DEGs in NC *vs* LPS comparison				
Source genes	Gene description	Regulation	Log2FC	p-value
*IL6*	Interleukin 6	Up	8.37	1.56E-11
*CD4*	CD4 antigen	Down	-1.61	0.0002
*CXCL10*	Chemokine (C-X-C motif) ligand 10	Up	4.91	2.67E-14
*CCL2*	Chemokine (C-C motif) ligand 2	Up	5.45	8.95E-23
*CSF2*	colony-stimulating factor 2	Up	2.88	0.006
*CXCL1*	Chemokine (C-X-C motif) ligand 1	Up	6.82	4.20E-20
*IL18*	Interleukin 18 receptor 1	Down	-1.16	2.01E-05
*CXCL9*	Chemokine (C-X-C motif) ligand 9	Up	3.37	8.87E-09
*MYD88*	Myeloid differentiation primary response gene 88	Up	2.3	2.72E-09
*ITGAM*	Integrin alpha M	Down	-1.05	0.0001
Top 10 hub genes of DEGs in LPS *vs* LPS+MDP comparison				
*CD74*	CD74 antigen (invariant polypeptide of major histocompatibility complex, class II antigen-associated)	Up	1.86	2.20E-07
*CIITA*	Class II trans-activator	Up	2.15	8.89E-24
*H2-AB1*	Histocompatibility 2, class II antigen A, beta 1	Up	1.63	1.30E-07
*H2-DMA*	Histocompatibility 2, class II, locus DMa	Up	2.22	6.67E-14
*H2-AA*	Histocompatibility 2, class II antigen A, alpha	Up	1.76	3.65E-07
*H2-DMB1*	Histocompatibility 2, class II, locus Mb1	Up	2.85	6.57E-21
*H2-EB1*	Histocompatibility 2, class II antigen E beta	Up	1.66	1.67E-07
*DEFA21*	Defensin, alpha, 21	Up	7.69	7.19E-17
*ITGAM*	Integrin alpha M	Up	1.95	5.02E-13
*DEFA5*	Defensin, alpha, 5	Up	4.97	1.77E-07

### 3.6 qRT-PCR confirmation of the DEGs

**Figure 6:**
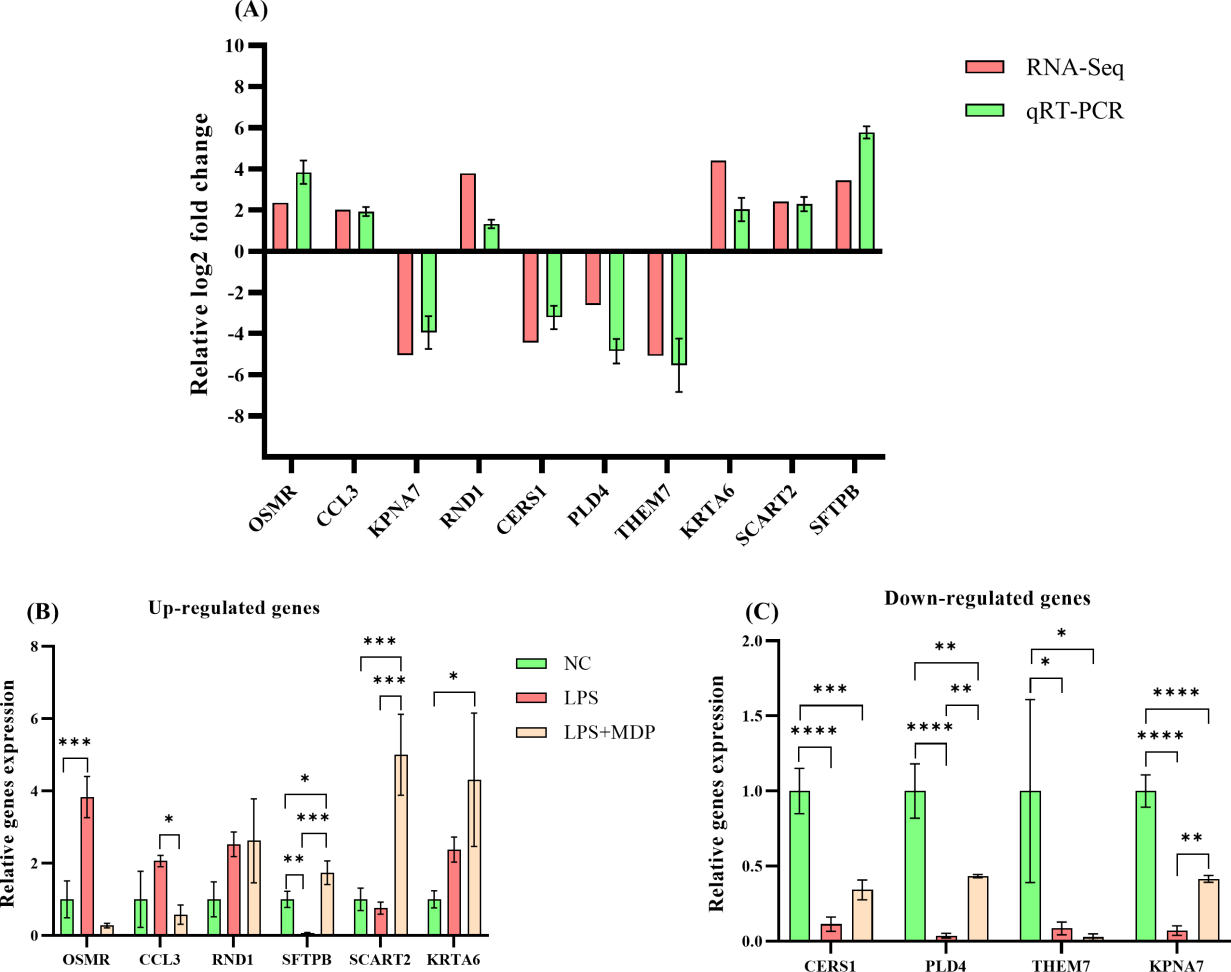
The qRT-PCR validation of RNA-seq results and differential expression analysis of selected genes. The relative log_2_ fold change (log_2_FC) of gene expression is plotted on the y-axis, with individual genes represented on the x-axis. Genes that are upregulated are positioned above the horizontal reference line, while downregulated genes appear below it **(A)**. The qRT-PCR analysis of selected differentially expressed genes (DEGs) across three groups: NC, LPS, and LPS+MDP. Data are expressed as means ± standard deviation (SD) from three independent experiments. Statistical significance was determined using Student’s t-test for comparisons between the groups, with p-values indicated as follows: ****P < 0.0001, ***P < 0.001, **P < 0.01, *P < 0.05. The x-axis represents the relative gene expression levels, and the y-axis shows individual genes. **(B)** displays genes that are upregulated, while **(C)** shows genes that are downregulated.

To validate the accuracy of our RNA-sequencing results, qRT-PCR was employed to validate the expression of 10 differentially expressed genes (DEGs) selected randomly, thereby confirming the accuracy of the RNA-seq results in this study. The expression patterns of all validated genes were consistent with those observed in the sequencing data (**Figure 6A**). Notably, 6 genes were significantly upregulated (**Figure 6B**), and 4 genes were significantly downregulated in the treatment group relative to the control (**Figure 6C**), further underscoring the robustness and reliability of the transcriptome data. These findings demonstrate that the RNA-seq analysis accurately reflected the transcriptomic changes occurring in the system (**Figure 6**).

### 3.7 Impact of MDP on microbial diversity and community structure

**Figure 7:**
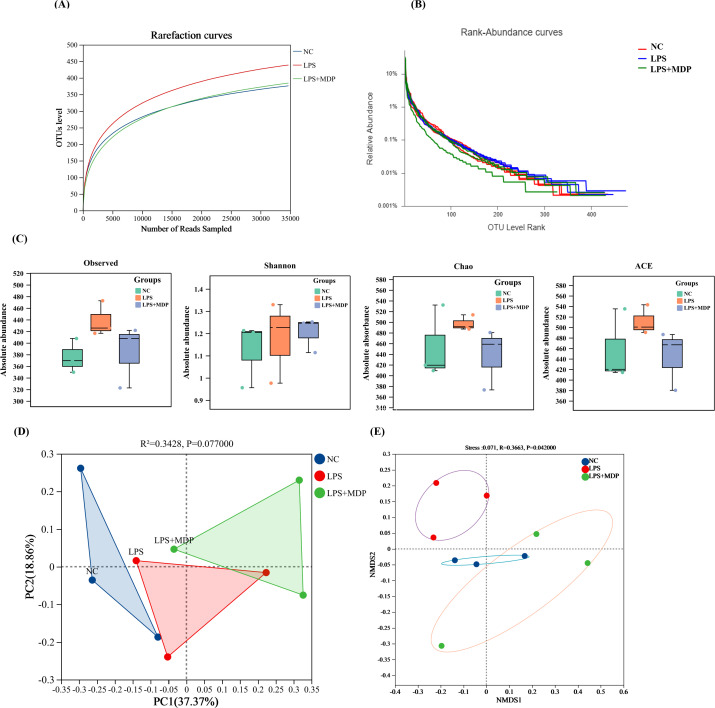
Rarefaction and the rank abundance curves of OTUs **(A, B)**. The Alpha Diversity: Box plots illustrate the alpha diversity of the microbial communities, including four different metrics: Observed OTUs, Shannon Index, Chao, and ACE **(C)**. PCoA plot **(D)**, and Non-metric Multidimensional Scaling (NMDS) plots showing the beta diversity of the microbial communities across samples **(E)**.

To further assess the impact of MDP treatment on microbial diversity, we calculated rarefaction, and rank abundance curves, alpha and beta diversity indices, including Observed, Shannon, ACE (Abundance-based Coverage Estimator), and Chao, PoCA, and NMSD diversity indices respectively (**Figure 7**). **Figures 7A, B** present the rarefaction and rank abundance curves for the three experimental groups, providing insights into the sequencing depth and richness of microbial communities under different treatments. Based on the rarefaction optimum species saturation was achieved, indicating that the sample size was adequate for accurately assessing bacterial diversity. The rank abundance curves showed that the OTUs differed greatly in abundance, and had low uniformity in their distribution within each sample. **Figure 7C** illustrates how the alpha diversity metrics differ across the three experimental groups. The LPS+MDP group exhibited notable shifts in these indices compared to the NC and LPS groups, suggesting that MDP treatment influences not only the overall diversity but also the structure of the microbial communities. This indicates that MDP may have a significant effect on the microbial population’s resilience and composition following LPS-induced inflammation. Furthermore, the results of the PERMANOVA analysis using the unweighted Unifarc distance (R^2^ = 0.3428, P=0.077) revealed the differences between the microbial communities of the NC, LPS, and LPS+MDP groups (**Figure 7D**). These findings underscore the impact of MDP treatment on the microbial community composition, with MDP-induced shifts in community structure compared to the LPS and control groups. The Unweighted Unifrac distances provided strong evidence that MDP treatment can lead to substantial changes in the microbial community, which may be linked to its anti-inflammatory effects or its ability to modulate the gut microbiome in response to LPS-induced inflammation. Furthermore, the NMDS plot based on the Bray-Curtis distance (R^2^ = 0.3663, P=0.042) revealed clear differences among the NC, LPS, and LPS+MDP groups (**Figure 7E**) These results indicated that MDP treatment is linked to a significant alteration in microbial community composition.

### 3.8 MDP supplementation ameliorates LPS-induced changes in gut microbial abundance and taxonomic profiling

**Figure 8:**
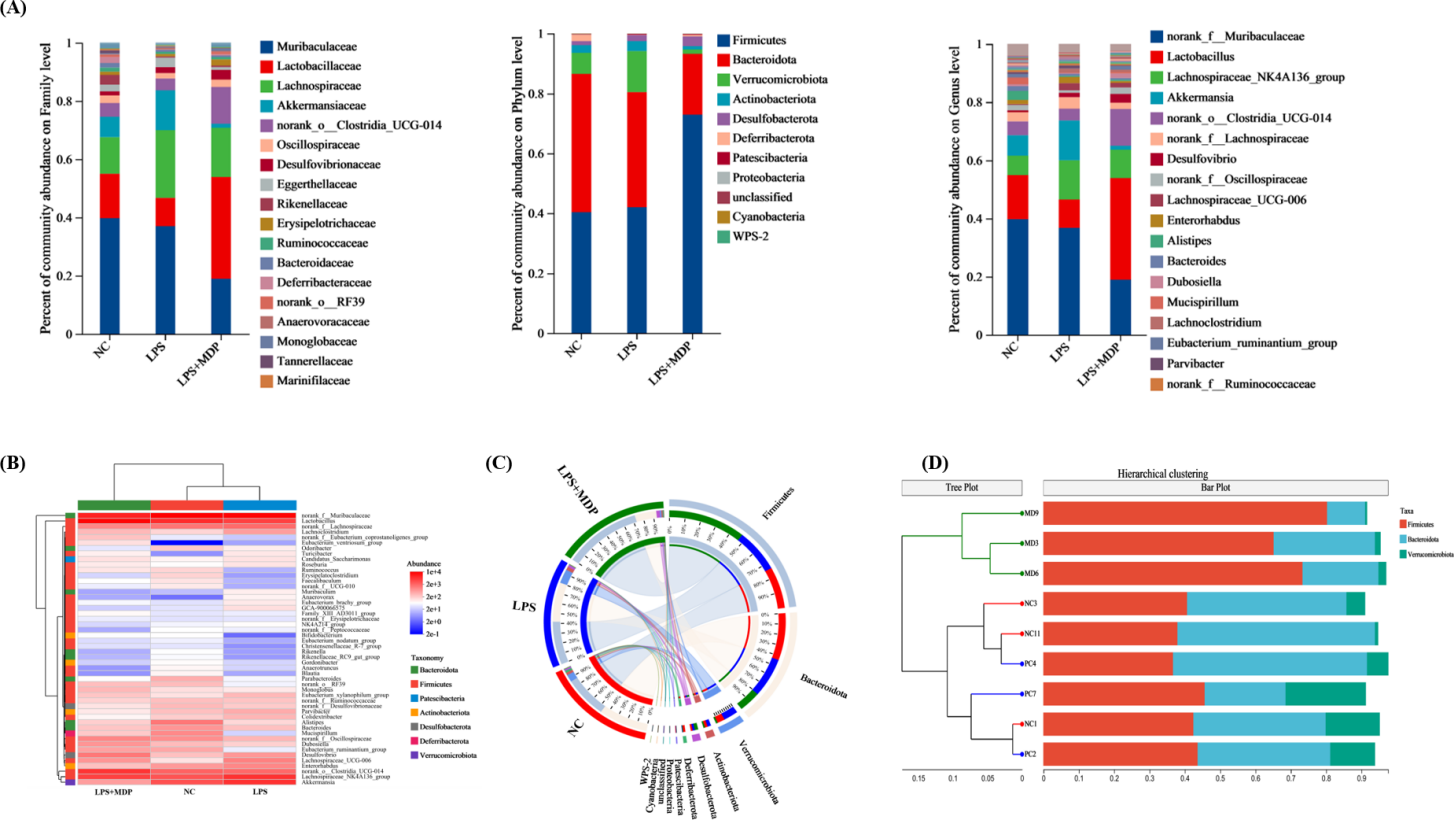
The microbial distribution in the control, LPS, and LPS+MDP groups. Bar plots showing the relative abundance of microbial taxa at the phylum, family, and genus levels **(A)**. The heatmap shows the distribution of the top dominant species **(B)**, Circo’s analysis at the OTU level **(C)**, and sample hierarchical cluster tree **(D)**.

We examined 16S RNA gene sequences from 9 fecal samples across 3 groups (NC, LPS, and LPS+MDP) to assess changes in microbial communities following MDP treatment (**Figure 8**). The changes in the gut microbiota across different taxonomic levels (phylum, family, and genus) for all groups are shown in **Figures 8A-D**. The taxonomic analysis revealed that the microbial communities in all three groups were primarily dominated by three major phyla: *Firmicutes*, *Bacteroidetes*, and *Verrucomicrobiota*. These phyla accounted for the largest proportion of the microbiome across the samples, with relative abundances varying. Furthermore, we observed that three predominant phyla including Firmicutes showed an abundance of (40.36-72.93%), *Bacteroidetes* (20.32-46.16%), and *Verrucomicrobiota* (1.3-21.27%) (**Figure 8A**). Among these, *Firmicutes* emerged as the predominant phylum, especially in the LPS+MDP group, where its abundance was significantly higher compared to the other groups. In contrast, the *Bacteroidetes* phylum was more prevalent in the NC group, suggesting that the LPS-induced inflammation and subsequent MDP treatment could alter the relative abundance of these key microbial groups. The heat map visually displayed the distribution of the top dominant species in different groups in all samples and explored the species change trend between the control group and the treatment group (**Figure 8B**). These microbiota alterations were also confirmed by Circo’s analysis at the OTU level, where distinct bacterial populations were observed among the different groups (**Figure 8C**). Moreover, the sample hierarchical cluster tree was used to show the similarities and differences in species composition among samples, and combined with the stacked column chart, the overall composition differences of samples and the distribution of species composition among different samples were more intuitively displayed. Specifically, in the LPS group, there was a decrease in the proportion of *Firmicutes* and an increase in the proportion of *Bacteroidetes* compared to the NC mice (**Supplementary Table S2**). However, MDP supplementation significantly reversed these shifts in bacterial proportions in the LPS-induced inflammatory mice.

## 4 Discussion

In this study, we investigated the protective effects of mulberry-derived postbiotics on inflammation in LPS-induced mice, with a particular focus on their impact on the transcriptomic landscape and gut microbiome. Our results provide compelling evidence that these postbiotics modulate key molecular pathways associated with immune response and inflammatory regulation. Specifically, we observed significant alterations in gene expression and microbiome composition that correlate with reduced inflammatory markers and improved immune function. The intestinal mucosa is essential for defending against harmful substances and regulating immune responses (43, 44). This barrier, maintained by tight junctions and supported by immune cells in the lamina propria, is disrupted in conditions like LPS-induced endotoxemia, leading to impaired intestinal integrity (45). In our study, LPS treatment caused significant damage to the intestinal architecture, including villous atrophy and reduced villus height and crypt depth, which are indicators of compromised barrier function (46). The disruption of the villus-crypt ratio (V/C) further confirmed epithelial stress and a failure to maintain homeostasis (47). Interestingly, pretreatment with mulberry-derived postbiotics (MDP) mitigated these histological changes, restoring villus length, and crypt depth, and normalizing the V/C ratio in the LPS+MDP group. These results suggest that MDP offers protective effects on the intestinal mucosa, aiding recovery from LPS-induced damage. Previous studies have shown that postbiotics promote mucosal healing by enhancing epithelial cell turnover, reducing inflammation, and restoring tight junctions (23, 48). The present findings are consistent with these studies, indicating that MDP may play a key role in maintaining or restoring the function of the intestinal barrier in the context of inflammatory insults. Recent studies further demonstrate that postbiotics can enhance mucosal barrier integrity by promoting the proliferation of epithelial cells and increasing the expression of tight junction proteins, which helps restore intestinal permeability compromised during inflammation (49, 50). The Jejunum was specifically chosen for this analysis due to its crucial role in nutrient absorption and its accessibility for studying early microbial and immune interactions, despite the fact that microbial activity predominantly occurs in the colon and ileum (51). This choice is supported by the previous work that highlights the jejunum’s involvement in immune responses and the modulation of gut microbiota even in the presence of significant microbial activity further downstream in the digestive tract (52). Furthermore, the jejunum is an important site for the initial immune responses to microbial disturbances and has been shown to exhibit a high density of immune cells that can influence systemic inflammation (53). While the colon and ileum are indeed more heavily populated by microbiota, the jejunum provides valuable insights into the early events of immune modulation and microbial interactions (52), which are key in understanding the overall therapeutic impact of MDP.

The results from the differential expression analysis and functional enrichment suggest that LPS treatment induces a robust immune response, activating key biological processes such as immune system regulation, cellular signaling, and responses to stimuli. These findings are consistent with previous studies showing that LPS, a potent bacterial endotoxin, triggers immune activation through the Toll-like receptor 4 (TLR4) pathway, which plays a critical role in inflammation and host defense mechanisms (54, 55). The observed enrichment of genes involved in immune processes, such as *IL6*, *CXCL10*, and *CCL2*, further supports the notion that LPS predominantly activates inflammatory responses. Interestingly, when comparing the LPS and LPS+MDP treatments, the overlap in enriched biological processes suggests that MDP does not introduce fundamentally new immune pathways but rather modulates the existing LPS-induced immune response. KEGG pathway analysis further supports these observations, identifying several key pathways associated with immune regulation, stress responses, and antimicrobial activity. Both the NC *vs* LPS and LPS *vs* LPS+MDP comparisons revealed significant enrichment in pathways related to immune responses, cellular defense, and responses to external stimuli. These pathways are essential for the host’s defense against infections and in managing systemic stress (56). While many of the pathways activated by LPS remained prominent, the addition of MDP influenced the dynamics of immune modulation. Notably, the enrichment in antigen processing and presentation pathways suggests that MDP may enhance the adaptive immune response, potentially facilitating a more targeted resolution of inflammation. This contrasts with the broader, nonspecific inflammatory activation observed in LPS treatment alone. Furthermore, the persistent enrichment in NOD-like receptor signaling indicates that MDP may refine the innate immune response, ensuring a more controlled activation of inflammatory pathways without overwhelming the system, as seen in the LPS group.

The pathways related to transcriptional misregulation observed in both LPS and LPS+MDP groups suggest that MDP may play a role in stabilizing gene expression patterns disrupted by LPS, potentially reducing the overexpression of pro-inflammatory cytokines and other mediators that contribute to chronic inflammation. By modulating transcriptional networks, MDP could restore cellular homeostasis and reduce the prolonged inflammatory response triggered by LPS. This suggests that MDP could be a potential modulator of immune responses, particularly in conditions where fine-tuning the immune system is crucial for optimal function.

Hub genes identified through protein-protein interaction (PPI) network analysis, such as *IL6*, *CXCL10*, *MYD88*, and *ITGAM* in the NC *vs* LPS group, represent central regulatory nodes in immune signaling and inflammation. IL6 and CXCL10, for instance, are well-known mediators of immune cell recruitment and inflammatory cascades (57). In the comparison between LPS and LPS+MDP, genes like *CD74*, *CIITA*, and *HLA*-related genes (*H2-AB1*, *H2-DMA*) emerged as central players, emphasizing the role of antigen presentation in the adaptive immune response. CD74 and CIITA are involved in the major histocompatibility complex (MHC) class II pathway, suggesting that MDP enhances antigen presentation and thus bolsters adaptive immunity (58, 59). Recent studies have also shown that postbiotics, particularly those from the *Lactobacillus* genus, can enhance antigen presentation by increasing MHC class II expression on antigen-presenting cells, thereby improving adaptive immunity in inflammatory settings (25). The identification of these hub genes provides a valuable target list for further research into the mechanisms through which MDP modulates both innate and adaptive immune responses. Collectively, these findings highlight that MDP’s primary role is likely to optimize immune responses initiated by LPS, rather than altering the fundamental immune pathways involved.

Our results revealed significant shifts in microbial diversity following MDP treatment. We observed that *Firmicutes* and *Bacteroidetes* dominated the microbial communities, with *Firmicutes* being more abundant in the LPS+MDP group and *Bacteroidetes* prevalent in the NC group. These results are consistent with previous studies linking *Firmicutes* to conditions of dysbiosis and *Bacteroidetes* to a balanced gut microbiota (60, 61). Additionally, *Verrucomicrobiota* was more abundant in the LPS and LPS+MDP groups, suggesting that MDP may promote the growth of beneficial microbes, such as *Akkermansia muciniphila*, known for its role in gut health and immune modulation (62, 63). Recent literature supports this finding, with studies indicating that postbiotics derived from various probiotic strains can selectively promote beneficial microbes, such as *Akkermansia muciniphila*, which has been associated with improvements in gut barrier function and reduction of inflammatory markers in the gut (64). The diversity indices (Shannon, ACE, Chao) illustrated significant recovery of microbial diversity in the LPS+MDP group, aligning with findings that microbial diversity is often reduced under inflammatory conditions and that interventions like MDP can restore this balance (65). PERMANOVA analysis further confirmed that MDP treatment significantly altered microbial composition, underscoring its potential therapeutic role in modulating gut microbiota in inflammatory contexts. This restoration of microbial diversity is in line with emerging evidence that postbiotics can help re-establish microbial balance in the gut, leading to improvements in intestinal health and immune function (66).

In this study, we observed that mulberry-derived postbiotics (MDP) modulated both immune pathways and the gut microbiome in LPS-induced inflammation, suggesting a synergistic interaction between host gene expression and microbial composition. Key hub genes such as *IL6*, *CXCL10*, and *MYD88* were significantly altered in the LPS-treated mice, indicating a robust immune response. Specifically, IL6 and CXCL10 are inflammatory cytokines that recruit immune cells and trigger inflammatory cascades (67, 68). In the LPS+MDP group, these pro-inflammatory markers were downregulated, correlating with the restoration of intestinal barrier integrity and reduced inflammation. This modulation of immune signaling by MDP could be linked to shifts in microbial populations, particularly the increase in *Firmicutes* and *Verrucomicrobiota*, which are associated with anti-inflammatory effects and immune regulation (69, 70). For instance, *Akkermansia muciniphila*, a key member of Verrucomicrobiota, has been shown to enhance mucosal barrier function and modulate immune responses via Toll-like receptor (TLR) signaling (71). This aligns with the reduction in *CXCL10*, as *Akkermansia* promotes tolerance and reduces systemic inflammation.

Additionally, genes involved in antigen presentation, such as *CD74* and *CIITA* (72), were upregulated in the LPS+MDP group, suggesting that MDP enhances adaptive immunity. *Bacteroidetes*, particularly *Bacteroides fragilis*, which increased in the LPS+MDP group, are known to promote immune tolerance and regulate T regulatory cells (Tregs) (73, 74), further supporting the immune-modulatory effects of MDP. These microbial shifts likely contribute to the observed improvements in immune homeostasis, as *Bacteroidetes* species help resolve inflammation and maintain balance in immune responses (75, 76). *Firmicutes*, which became more abundant in the LPS+MDP group, are known to regulate immune responses, likely through their role in maintaining intestinal health and modulating systemic inflammation (38). Taken together, the transcriptomic and microbial data indicate that MDP’s therapeutic effects are mediated by both direct immune modulation and the restoration of a balanced gut microbiome, which works synergistically to reduce inflammation and improve gut health. Taken together, the transcriptomic and microbial data indicate that MDP’s therapeutic effects are mediated by both direct immune modulation and the restoration of a balanced gut microbiome, which works synergistically to reduce inflammation and improve gut health. These findings underscore the importance of the microbiome-immune axis in regulating inflammatory responses and highlight MDP as a potential therapeutic strategy for managing inflammatory diseases. The integration of transcriptomics analysis provides vital insights into specific pathways modulated by MDP, enabling the identification of key molecular targets for further therapeutic interventions. Moreover, the broad implications of these finding suggests that the implementation of MDP could extend beyond gastrointestinal health, potentially benefiting individuals with systemic inflammatory conditions. The ability of these postbiotics to enhance beneficial microbial populations while suppressing pathogenic ones may also pave the way for personalized microbiome targeted therapies, aligning with contemporary approaches to precision medicine. Future studies should focus on validating these findings in clinical settings and exploring the long-term effect and safety profiles of MDP. Furthermore, examining the synergistic effects of these MDP with traditional treatments may yield enhanced therapeutic outcomes for patients suffering with chronic inflammatory diseases.

## 5 Conclusion

In conclusion, this study provides evidence that mulberry-derived postbiotics (MDP) exert protective effects in LPS-induced inflammation by ameliorating histological damage to the intestinal mucosa, restoring key architectural features such as villus length, crypt depth, and the villus-crypt ratio. Transcriptomic analysis revealed that MDP modulated the LPS-induced immune response by influencing pre-existing immune pathways, rather than introducing novel pathways. Notably, MDP enhanced antigen presentation and adaptive immunity through the regulation of hub genes involved in immune signaling. Additionally, MDP treatment resulted in a shift in microbial diversity, promoting the expansion of beneficial bacteria, including *Akkermansia muciniphila*, known for its roles in gut health and immune modulation. These findings suggest that MDP holds therapeutic potential for restoring immune homeostasis and gut microbiota balance in inflammatory conditions, making it a promising strategy for managing dysbiosis and enhancing gut health.

## Data Availability

The original contributions presented in the study are deposited in the NCBI repository. This data can be found here: PRJNA1224036 (Microbiome Data) and PRJNA1224420 (Transcriptomics Data).
