# Supplementary material for: Transcriptomics and microbiome insights reveal the protective mechanism of mulberry-derived postbiotics against inflammation in LPS-induced mice
Source: Front Immunol. 2025 Feb 18;16:1536694. doi: 10.3389/fimmu.2025.1536694 (PMC11876837; doi:10.3389/fimmu.2025.1536694)
Supplement: Supplementary file 1 [file DataSheet1.docx]

**Mulberry-derived Postbiotics Alleviates LPS-induced Intestinal Inflammation and Modulate Gut Microbiota Dysbiosis**

**Zaheer Abbas ^1^, Baseer Ahmad ^2^, Yucui Tong ^1^, Zhang Jing ^1^, Shuang Wu ^1^, Junyong Wang ^1^, Zhenzhen Li ^1^, Tianqi Liu ^1^, Yicong Liu ^1^, Xubioa Wei ^1^, and Dayong Si ^1*^, and Rijun Zhang ^1*^**

1. State Key Laboratory of Animal Nutrition and Feeding, College of Animal Science and Technology, China Agricultural University, 100193, Beijing, China; [zaheerabbas@cau.edu.cn](mailto:zaheerabbas@cau.edu.cn)
2. Faculty of Veterinary and Animal Science, Muhammad Nawaz Sharif University of Agriculture, Multan, 25000, Pakistan.

***** Correspondence: [dayong@cau.edu.cn](mailto:rjzhang@cau.edu.cn), [rjzhang@cau.edu.cn](mailto:rjzhang@cau.edu.cn)


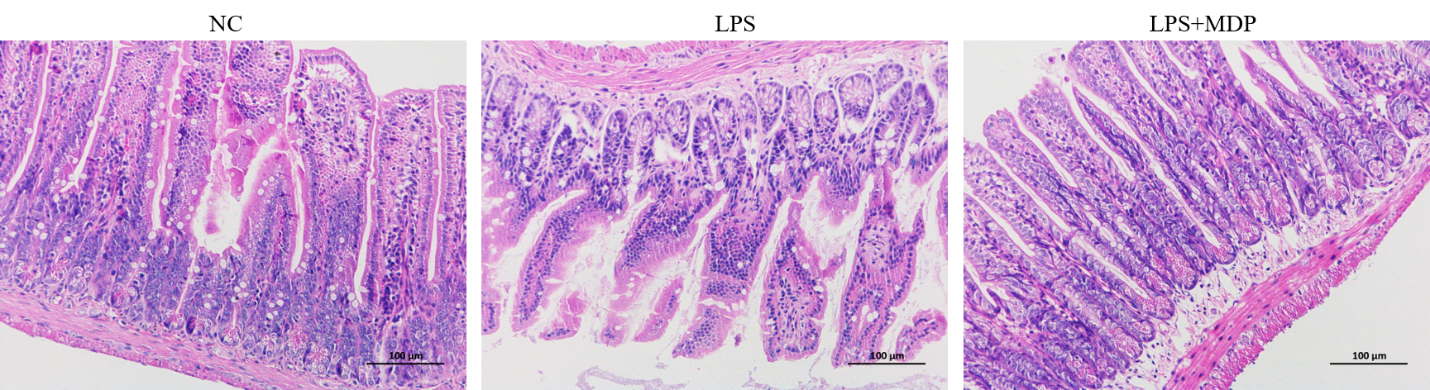


Fig S1. H&E staining images (100 µm) of jejunum tissue from negative control (NC), positive control (LPS), and treatment (LPS+MDP) groups.

Table S1. Primer used for the qRT-PCR in this study

| **Gene** | **Primer sequence** | **Primer length** |
| --- | --- | --- |
| *OSMR* | F: 5’-GGAAATACCTTGGCAGTGGTG | 21, 21 |
|  | R: 5’-GCTACCAAACCTCGGTAATCC |  |
| *CCL3* | F: 5′-CCAAGTCTTCTCAGCGCCAT 3′ | 20, 20 |
|  | R: 5′-TCCGGCTGTAGGAGAAGCAG 3′ |  |
| *Rnd1* | F: 5′-GGAGACAGAGGAACAGAGGGTG-3’ | 22, 20 |
|  | R: 5‘-CGGGTGCTGGGACAATAATC-3’ |  |
| *CERS1* | F: 5′-TGGTTCCTGTACATCGTGGC-3′ | 20, 20 |
|  | R: 5′-CTCAGTGGCTTCTCGGCTTT-3′ |  |
| *PLD4* | F: 5′-GACTGGAGTTCCCACTATGCTAT-3′ | 20, 20 |
|  | R: 5′-AGGTGGCAGGGTTTTATTGTGGCT-3′ |  |
| *Them7* | F: 5′-GCAGGACAATGACCAGAATAGCC-3′ | 25, 22 |
|  | R: 5′-GTCAGCAAGCTGGTTTTCAGGC-3′ |  |
| *SFTPB* | F: 5′-CCAAGTGCTTGATGTCTACC-3′ | 20, 20 |
|  | R: 5′-CTGGATTCTGTTCTGGCTTA-3′ |  |
| *Scart2* | F: 5′-GGTTGGCAGCGGGTAAGAAC-3′ | 20, 19 |
|  | R: 5′-AATCGTAGACGAGCCCCTT-3′ |  |
| *Krt6a* | F: 5′-AATCGATCCCACCATCCAGC-3′ | 20, 20 |
|  | R: 5′-CTCCAGGTTCTGCCTCACAG-3′ |  |
| *Myo16* | F: 5′-ATGGAAATTGACCAGTGCTTGCTGGA-3′ | 26, 24 |
|  | R: 5′-CTGTCAATGAGGACTTCTGCGATG-3′ |  |
| *KPNA7* | F: 5′-CATCGAGAAGCACTTTGGTG-3′ | 20, 20 |
|  | R: 5′-GGAGGTAGGGAGCTTGGCTA-3′ |  |
| *Chrna2* | F: 5′-GTAGATCTCCGCGCAGCAGTC-3' | 20, 20 |
|  | R: 5′-AGTGCAGACGGGGAGTTTGCG-3' |  |

Table S2 shows the relative abundance of the top gut microbiota on the phylum, family and genus level in each sample.

| **Relative abundance (%)** | **NC** | **LPS** | **LPS+MDP** |
| --- | --- | --- | --- |
| **Phylum** |  |  |  |
| Firmicutes | 0.40±0.02 | 0.42±0.04 | 0.46±0.09 |
| Bacteroidetes | 0.46±0.09 | 0.38±0.16 | 0.38±0.13 |
| **Family** | | | |
| Muribaculaceae | 0.39±.05 | 0.37±0.14 | 0.40±.07 |
| Lactobacillaceae | 0.15±0.05 | 0.09±0.02 | 0.21±.06 |
| Lachnospiraceae | 0.12±0.01 | 0.23±0.06 | 0.16±0.10 |
| **Genus** | | | |
| norank_f__Muribaculaceae | 0.39±0.05 | 0.36±0.14 | 0.40±0.06 |
| Lactobacillus | 0.15±0.05 | 0.09±0.02 | 0.17±0.12 |
| Akkermansia | 0.07±0.07 | 0.13±0.07 | 0.05±0.03 |
| Lachnospiraceae_NK4A136 | 0.06±0.01 | 0.13±0.03 | 0.07±.09 |
